# Neighborhood characteristics as determinants of healthcare utilization – a theoretical model

**DOI:** 10.1186/s13561-019-0226-x

**Published:** 2019-03-06

**Authors:** Sigrid M. Mohnen, Sven Schneider, Mariël Droomers

**Affiliations:** 1 National Institute for Public Health and the Environment (RIVM), Centre for Nutrition, Prevention, and Health Services, PO Box 1, 3720 BA Bilthoven, The Netherlands; 2 Mannheim Institute of Public Health, Social and Preventive Medicine (MIPH), Heidelberg University, Mannheim, Germany; 3 Utrecht Municipality, Department of Public Health, PO Box 16200, 3500 CE Utrecht, The Netherlands

**Keywords:** Risk assessment, Prediction, Andersen model, Regional, Sociology, A120 Relation of Economics to Other Disciplines, I110 Analysis of Health Care Markets, G220 Insurance; Insurance Companies; Actuarial Studies, I130 Health Insurance, Public and Private, I180 Health: Government Policy; Regulation; Public Health, R00 General (Urban, Rural, Regional, Real Estate, and Transportation Economics)

## Abstract

**Background:**

We propose using neighborhood characteristics as demand-related morbidity adjusters to improve prediction models such as the risk equalization model.

**Results:**

Since the neighborhood has no explicit ‘place’ in healthcare demand models, we have developed the “Neighborhood and healthcare utilization model” to show how neighborhoods matter in healthcare utilization. Neighborhood may affect healthcare utilization via (1) the supply-side, (2) need, and (3) demand for healthcare – irrespective of need. Three pathways are examined in detail to explain how neighborhood characteristics influence healthcare utilization via need: the physiological, psychological and behavioral pathways. We underpin this theoretical model with literature on all relevant neighborhood characteristics relating to health and healthcare utilization.

**Conclusion:**

Potential neighborhood characteristics for the risk equalization model include the degree of urbanization, public and open space, resources and facilities, green and blue space, environmental noise, air pollution, social capital, crime and violence, socioeconomic status, stability, and ethnic composition. Air pollution has already been successfully tested as an important predictive variable in a healthcare risk equalization model, and it might be opportune to add more neighborhood characteristics.

**Electronic supplementary material:**

The online version of this article (10.1186/s13561-019-0226-x) contains supplementary material, which is available to authorized users.

## Background

It is not only individual characteristics on the micro level that are relevant to an individual’s demand for healthcare, but also the meso level: the physical and social environment in which a person lives, the local neighborhood. We propose applying neighborhood characteristic as morbidity adjusters to improve prediction models on healthcare utilization. Based on the rich literature on neighborhood health effects, we assume that neighborhood characteristics, i.e. the small-area environment a person lives in, affect health [1–3], and since health is considered to be the major determinant of healthcare demand, we assume that taking account of neighborhood characteristics could improve the accuracy of models that seek to predict healthcare utilization.

This improvement may be due to the fact that unexplained geographic variations in healthcare utilization have been demonstrated, even when individual characteristics are taken into account [4]. Finkelstein et al. [5], who studied elderly persons who moved house, concluded that half of the geographic variation in healthcare utilization is due to place-specific factors. Most research on ‘geographic variation’ or ‘practice variation’ has focused on larger areas than neighborhoods [6], as well as on the supply-side as a cause for the variations between places [7, 8]. In this article, we propose that in addition to the supply-side, demand-related characteristics of the neighborhood play a role in variations in healthcare utilization. Hence, we aim to define characteristics of the neighborhood that are relevant to demand but independent of the supply side. Opposed to data-driven approaches, we present theory based suggestions for neighborhood characteristics relevant for health care prediction.

Some health economists have already acknowledged the potential of ‘traditional environmental factors’ in healthcare demand research [9, 10]. Erbsland et al. (1995) showed that ‘environmental pollution’ indirectly affects healthcare utilization via the stock of health capital. Van der Ven and Van der Gaag (1982) reported that the “percentage unemployed in a Dutch province is negatively associated with health” and the authors suggested that more exogenous variables should be included in the healthcare demand equation, such as “environmental hygiene, welfare work, sporting facilities.” (p 23). To our knowledge, however, a theoretically based overview of the influence of the neighborhood on healthcare utilization and its potential to improve the prediction of healthcare usage has so far been lacking.

To address this gap, we study the ‘place’ of the neighborhood in healthcare demand models and explain that the meso level (the neighborhood) is relevant to healthcare utilization in the “Neighborhood and healthcare utilization model” (Section Results). We underpin this theoretical model with literature on all relevant neighborhood characteristics relating to health and healthcare utilization (Section Results). In Section Discussion, we elaborate on the idea of using neighborhood characteristics to predict healthcare utilization as well as healthcare spending.

## Methods

We have developed a conceptual model, visualized it in a figure and gave a detailed description of each part of the model. Here, we are building on achievements in neighborhood health research that include disciplines such as health geography, sociology, epidemiology, and environmental research. Moreover, we present mechanisms and pathways linking parts of the model, which enables the reader to understand why neighborhood characteristics might affect health care demand.

In order to generate hypotheses – based not only on theory but also on empirical evidence – regarding neighborhood characteristics as predictors for healthcare utilization, we have summarized the literature in Section List of neighborhood characteristics relevant for health care utilization and presented it in the Additional files 1 and 2. This overview provides an indication of which neighborhood characteristics could potentially affect healthcare utilization, although it is not intended to be an exhaustive summary or a systematic review. We relied on reviews wherever possible, and where reviews were not available, we made use of individual papers from peer-reviewed scientific journals. As most relevant studies are cross-sectional in nature, our aim is to generate hypothesis. Furthermore, the definition of neighborhood varies considerably, including areas as big as large cities [11]. Morbley et al. showed that studies using only county-level contextual factors miss some meaningful associations related to interpersonal/proximate-level factors. We therefore cannot guarantee that the cited articles all suit the mechanisms and pathways described in this paper, because of differences in geographical units. All the neighborhood characteristics presented are ‘public goods’, so we do not include individual environmental exposure, such as indoor air pollution, residents’ private pools or playgrounds, or indoor housing conditions.

The rows of the tables in the Additional files 1 and 2 include specific physical and social neighborhood characteristics, as well as a range of keywords derived from the literature referenced. The first column of study outcomes regards mediators. The mediators were health-related behaviors as eating habits, stress level, participation, and willingness to use healthcare. Study outcomes regarding need have been divided into two columns; one is for self-perceived or psychological outcome measures like self-perceived health, well-being, and self-perceived or diagnosed mental health; the other is for physical health outcome measures like disease and mortality. Finally, the last column relates to healthcare utilization (e.g., use of medication or preventive care, doctor visits, etc.). Some of the studies mentioned in this column looked at healthcare utilization as a proxy for disease, because direct health measures were not available. Therefore these studies did not set out to study the effect of neighborhood on healthcare utilization, but they did nevertheless report on the association between neighborhood characteristics and healthcare utilization - hence their inclusion here.

## Results

### Current healthcare demand models neglect the neighborhood

In order to discuss the role of the neighborhood in healthcare demand prediction models, we will first explain some terminology. We define ‘need’ as the necessity of a person to consume healthcare in order to maintain or restore physical and mental health. Need can be diagnosed by a professional (objective need), observed by the patient (subjective need) or remain unobserved. ‘Demand’ is defined as the objective requirement for healthcare in order to achieve or maintain good physical and mental health. Demand can also occur in the absence of need, such as in the case of preventive care, or without any health-related needs, as in the case of unnecessary care (patient-, or supplier-induced overuse). In addition to overuse, there is also underuse; not everybody who is in ‘need’ is aware of this, and even when the need is recognized, not everybody is willing to demand care. When demand finally leads to action, we talk about ‘healthcare utilization’, or just utilization.

To understand the processes that lead to healthcare utilization, it is necessary to study the mechanisms involved. Andersen’s model of healthcare demand [12] has been discussed and applied many times in order to explain utilization. This behavioral model, with healthcare utilization as its outcome variable, focuses on the individual behavioral processes that underlie the decision to consume healthcare or not, and hence mainly identifies individual characteristics that influence this decision. The main elements of the model are ‘predisposing factors’ ➔ ‘enabling factors’ ➔ ‘need’ ➔ and ‘utilization’ [13]. The impact of the external environment on health status or the need for healthcare was, however, acknowledged, although only in the third version of the model [14] and was again omitted from later versions. In the Andersen model, the external environment can affect utilization via two mechanisms. First, the neighborhood can directly affect healthcare utilization at the micro-level through *enabling* and, second, indirectly via *need*. Andersen and Newman ([13], page 16) define enabling as “Enabling conditions make health service resources available to the individual.” The neighborhood may vary in the nature of access to healthcare facilities, in terms of how many facilities are close and/or how easily they can be reached by transportation, for instance. Phillips et al. [15] worked with Andersen’s model to assess the use of environmental variables in the behavioral model of utilization. They defined the environment as characteristics of the healthcare delivery system, external environment, and community-level enabling factors. They concluded that the lack of environmental variables may have reflected confusion over the model’s conceptualization and that the environmental component may therefore be overlooked by many researchers [15]. Nonetheless, Verheij [16] applied the Andersen model to explain how the neighborhood affects utilization. He hypothesized that ‘urbanicity’ influences utilization via *need*, implying that a high degree of urbanization creates more need. These direct and indirect mechanisms are described further in the next section, in which we develop a healthcare utilization model with neighborhood characteristics as a central element and we explain the underlying mechanisms in order to understand the relationship between neighborhood and healthcare utilization.

### The neighborhood and healthcare utilization model

**Fig. 1:**
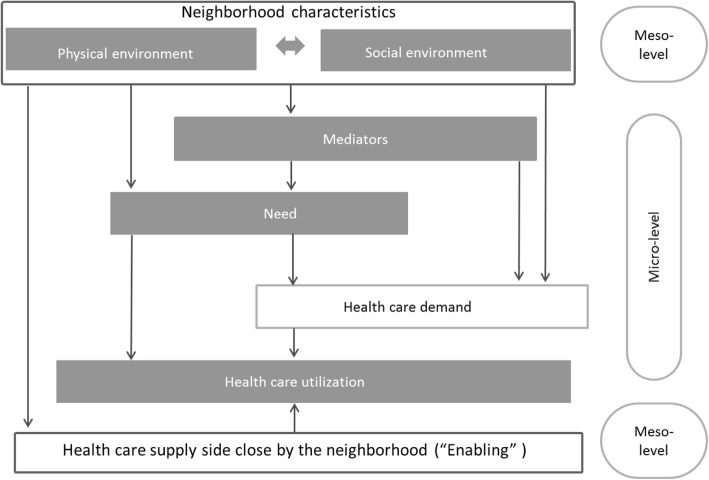
Neighborhood and healthcare utilization model

Figure 1 shows a simplified representation of the relationship between neighborhood at the meso level and healthcare utilization at the micro level. In this “Neighborhood and healthcare utilization model”, neighborhood characteristics may have positive or negative effects on healthcare utilization. The four arrows from the neighborhood characteristics indicate the mechanisms by which the neighborhood characteristics affect healthcare utilization. From left to right; the neighborhood can affect healthcare utilization via [1] the supply side, [2] via need, directly or through mediators, and via [3] demand for healthcare – irrespective of need. In this model, we will not address the converse influence of healthcare utilization on neighborhood characteristics, in order to keep the model focused.

In the following section, we will explain the elements of the model from meso- to micro-level, starting by defining neighborhood and neighborhood characteristics. We will then explain the mechanisms. The mechanism ‘Via need’ is divided into three subsections because the association of neighborhood characteristics via need to utilization can be explained by three different pathways. In section List of neighborhood characteristics relevant for health care utilization, we will use the dark grey filled boxes (these are the parts that can be operationalized) of the ‘Neighborhood and healthcare utilization model’ to structure the literature overview (Additional files 1 and 2).

### Definition of neighborhood and neighborhood characteristics

The idea that the neighborhood affects health is not new. Hippocrates, for instance, ascertained that diseases cluster geographically and he hypothesized that this phenomenon could be explained by ‘the local climate’ [17]. In the late nineteenth century, the physician John Snow came up with environmental explanations for the cholera outbreaks in certain neighborhoods in London. He plotted the cholera infections on a map of London and found an association with the water supply system. With no knowledge of the germ theory of diseases, he was able to identify the channel by which cholera spreads. Despite this, it was not until the end of the twentieth century that the ‘ecological approach’ placed people and their health back into context [18, 19]. The ecological approach in health research implies that human beings are social beings, living their lives in a certain context, not in a laboratory [20]. Moreover, environmental inputs that are relevant to health, such as pollution control, greater public safety, expanded opportunities to improve physical fitness, improved housing or access to education, are beyond the control of any one individual [21].

Since the emergence of the ecological approach, the neighborhood has been the context of many ecological studies on health outcomes [3]. Early studies focused on the variations in health between neighborhoods. These results widened the focus from individual determinants of health to the apparent impact of the living environment on individual health. The next wave of neighborhood health research studied the health impact of specific neighborhood characteristics. Two broad types of neighborhood characteristics are distinguished: physical and social [3]. Diez Roux and Mair state that the physical neighborhood characteristics include not only “traditional environmental exposures such as air pollution, but also aspects of the man-made built environment including land use and transportation, street design, other features of urban design and public spaces, and access to resources, such as healthy foods and recreational opportunities.” Rollings et al. [22] go into more detail in their summary of a full list of neighborhood physical attributes that could potentially be relevant to health: “land use, density, street connectivity, transportation availability and infrastructure, pedestrian and cycling infrastructure (presence, condition, and maintenance of sidewalks, bike lanes, cross walks, street lights, traffic lights); access to nature and green space, public and open spaces, and resources (public services, healthcare, healthy food, schools, playgrounds, commercial functions, and recreational opportunities); building and street condition, cleanliness, and maintenance; and traffic volume, air quality, and noise.” In addition to nature and green space, ‘blue space’ has also recently been considered relevant to health [23]. Social neighborhood characteristics include “the degree and nature of social connections between neighbors, the presence of social norms, levels of safety and violence, and various features of the social organization of places” [3]. Hereinafter, we also include socioeconomic aspects of the neighborhood in the social environment of the neighborhood (local prosperity and deprivation), as well as sociocultural aspects like the ethnic composition of a neighborhood community.

### Mechanisms

In order to understand how these neighborhood characteristics are able to affect healthcare utilization, we describe the mechanisms that may be responsible for a neighborhood effect on utilization, and we underpin these mechanisms with describing the underlying pathways.

#### ‘Supply-side’ -mechanism

As mentioned above, inspired by the Andersen’s model, the neighborhood is hypothesized to have an indirect effect on healthcare utilization via the supply-side (enabling), irrespective of actual need. Kawachi and Berkman [24] called it the ‘access to services and amenities’ pathway. Neighborhoods can differ in terms of distance, reachability, accessibility, as well as quantitative and qualitative characteristics of healthcare facilities [25]. In the US, people living in disadvantaged neighborhoods, irrespective of their individual-level characteristics, have reduced access to healthcare and were less likely to obtain recommended preventive service [26, 27]. A recent study on disadvantaged neighborhoods in Philadelphia did not reveal this association in relation to overall access, but living in a low-income neighborhood was associated with less reliance on physician’s offices and greater reliance on the safety net provided by health centers and outpatient clinics [28].

#### ‘Via need’ mechanism

‘Need’ can be operationalized as self-perceived health, well-being, mental health, diseases, and mortality. Neighborhood characteristics ‘get under the skin’ [29] via three different pathways; (1) physiological pathway, (2) psychological pathway, and (3) health behavioral pathway [30]. The first pathway is the direct effect of the neighborhood on health, while the two other pathways operate indirectly via mediators. Even though Berkman et al. formulated these pathways to explain the impact of the social environment on health, we believe that they also apply to the impact of physical neighborhood characteristics.

##### Physiological pathways between neighborhood and health

The physiological pathway derives from the field of biology and epidemiology and is represented by the arrow that connects neighborhood characteristics directly with need in Fig. 1. This pathway shows a dose response relationship: the stronger and/or longer the exposure to the neighborhood, the greater the effect on an individual’s health. In line with the ‘human basic need theory’ [31], a neighborhood protects health, because it provides the metabolic requirements for survival, which are basic physiological needs, such as air, water, food^1^ [32], shelter and security. Conversely, other physiological effects of the neighborhood can also damage health, such as polluted air, dirty water, nuclear radiation, or noise [33]. One example of a social neighborhood characteristic that has a direct physiological health effect is violent crime: a violent assault may lead to injury. Even when most basic needs are met, as in most Western countries, quality can vary between neighborhoods, leading to health variations between neighborhoods.

##### Psychological pathway between neighborhood and health

The neighborhood may affect health, on the one hand, through (the experience of) stress and, on the other hand, via the buffering effects of social support and social connections [3]. Chronic stress leads to increased levels of cortisol and other stress hormones, which adversely affects the immune system and increases the blood pressure and other biological risk factors for cardiovascular diseases and cancer [34]. Both physical and social neighborhood characteristics can induce stress. For example, the fear of crime and lack of safety can lead to stress, which negatively impacts mental and physical health [35]. The physical environment, too, such as the quality of the built environment, and the presence of traffic, noise, or a lack of resources, transportation, services, etc. have been linked to depression and other mental health problems [3].

In addition to inducing stress, physical and social neighborhood characteristics can also have just the opposite effect, helping to mitigate stress [3]. For example, contact with nature (e.g., green space) has short-term restorative effects [36] and is associated with good perceived mental health [37]. Social support also plays an important role in moderating reactivity in stressful situations [30]. For example, the social support generated in cohesive neighborhoods, particularly emotional support, has been shown to buffer the adverse effects of stressful life events on depression [30].

##### Health behavioral pathways between neighborhood and health

The neighborhood may also influence health through its impact on health-related behaviors, because it creates opportunities. Walkable, social, or safe neighborhoods provide more opportunities for physical activity (PA), and PA supports good health [38] and health-related quality of life [39]. The proximity of sales points for tobacco, alcohol and energy-rich food also influence health behaviors and thus ultimately affect health and healthcare demand among those who live in certain areas [40].

The neighborhood may also influence health-related behaviors through the presence of social norms and role models. Social cognitive theory states that individuals learn in social contexts; observing a neighborhood ‘model’ may influence individual behavior, and the same applies to the prevailing social norm towards certain behavior [41]. Social capital, defined as sharing common norms, behavioral reciprocity and mutual trust, varies between neighborhoods and has been associated with health-related behaviors, such as PA and smoking [42]. The same study did not support nutrition, sleep habits, or moderate alcohol intake as possible explanations of the effects of neighborhood-based social capital on health. Kawachi et al. [43] mention three possible pathways by which neighborhood-based social capital may influence health-related behavior: “[1] promoting more rapid diffusion of health information [44],^2^ [2] increasing the likelihood that healthy norms of behavior are adopted, and [3] exerting social control over deviant health-related behavior” ([43], page 1190).

Lastly, the neighborhood may influence health-related behaviors through the impact on future prospects. Deprived neighborhoods with high levels of crime and violence can influence the expected costs and benefits of adopting particular behaviors [45], since people who expect a shorter life-span are less motivated to prevent future health problems through, for example, quitting smoking [46] or avoiding alcohol abuse [25].

#### Healthcare demand mechanism

In addition to the effect of the neighborhood on health, we would like to draw attention to its effect on healthcare demand, irrespective of health, e.g. the demand for stop-smoking programs, even among those who are not suffering from smoking-related disease. The neighborhood may influence willingness to consume healthcare [25, 43]. For instance, the level of social capital, including social norms and values in a neighborhood may motivate people to seek out and use (preventive) healthcare, such as screening for colorectal cancer [47]. In neighborhoods with higher levels of social capital, information may be accessible and spread more easily ([43], page 1190). Information shared by word-of-mouth is usually taken more seriously and can make the difference in decisions to use healthcare – especially in case of preventive healthcare, thus in the absence of need or health problems. In addition to the information-dissemination pathway, Prentice [27] suggests that shared neighborhood health-behavior norms may lead to variation in healthcare demand. Another pathway could be ‘practical support from neighbors’ such as a ride to a doctor or taking care of children during medical visits [27].

### List of neighborhood characteristics relevant for health care utilization

To demonstrate the link between neighborhood characteristics and healthcare utilization, we present published scientific literature on neighborhood characteristics in relation to healthcare utilization, as well as to mediators and health outcomes, because need is an important mechanism in explaining the association between neighborhood characteristics and healthcare utilization (Additional files 1 and 2). All the dark grey boxes in Fig. 1 are used to structure Additional files 1 and 2. Additional file 1 shows physical neighborhood characteristics and Additional file 2 shows social neighborhood characteristics. Several studies reported on neighborhood characteristics in relation to healthcare utilization. However, no reviews were available about this association. The empirical evidence is much more detailed and diverse with regard to the association between neighborhood characteristics and health. Mediators of the association of neighborhood characteristics and health were also studied in great number. Additional files 1 and 2 therefore indicate that several neighborhood characteristics are likely to influence utilization; even so the evidence on utilization as a dependent variable is still thin.

Neighborhood characteristics that may influence healthcare utilization are degree of urbanization, public and open space, resources and facilities, green and blue space, environmental noise, air pollution (Additional file 1), social capital, crime and violence, socioeconomic status, stability of the neighborhood, and ethnic composition (Additional file 2). We found as many reviews on physical neighborhood characteristics as social neighborhood characteristics in relation to health. However, the studies on social environmental impacts are much more diverse (e.g. in terms of their definitions, indicators, measurement tools) and therefore do not lead neatly towards one conclusive summary, like reviews on more classical physical environmental hazards, such as the evidence on the impact of the health effects of air pollution.

Even so, most studies are of a cross-sectional nature and the well-known reporting bias that favors the publication of significant study outcomes may have caused an imbalance in our overview, we think that the association between neighborhood characteristics and utilization can be explained very well by combining Fig. 1 with Additional files 1 and 2. For example, neighborhoods with high levels of neighborhood ‘Crime and violence’ may be more likely than other neighborhoods to be perceived negatively by residents. Moreover, these neighborhoods might increasing stress levels and unhealthy behavior and low PA levels. It is therefore likely that neighborhood crime and violence weakens access to healthcare and willingness to demand healthcare, while the actual need to use healthcare may be higher than average.

## Discussion

Predicting healthcare utilization is part of the health services and payment models of most Western countries. An example of a prediction model is the *risk equalization model.* A risk equalization model equalizes the differences in financial risks between health insurers based on the characteristics of their clients [48]. A sophisticated and adequate risk equalization model (e.g., prediction based on morbidity adjusters is close to the true risk) can reduce the chance of risk selection by insurers, which increases the efficiency, quality and solidarity of the healthcare system [49]. Moreover, insurers should not be discouraged from preventing supplier-induced overuse, so supplier-related factors ought not be part of the equation. The need for utilization should be predicted irrespective of the access options and the kind of healthcare supplier.

Health status is therefore the central variable in healthcare demand models [50] and would be the preferred variable for predictions. Health status is, however, difficult to measure reliably and data is often not available for all individuals in a population. Therefore, predisposing variables that determine health are most often used as morbidity adjusters, as the second best option. Data on predisposing variables that can be used in prediction models is also rare, however, because it must be available for all the individuals of the population. For this reason, variables used hitherto in prediction models are likely to have been selected mainly because of their availability and less often because of their accuracy in predicting healthcare need and utilization. This data-driven approach may have led to a narrower perspective on the factors that affect (and thus predict) healthcare utilization. Healthcare utilization in previous years (prior utilization) has been used to substitute the missing information regarding health status to improve prediction models [51]. However, this comes at a price, because efficiency, quality and solidarity are not enhanced when (unreasonably high) prior utilization justifies (unreasonably high) utilization in the future, irrespective of actual health status. Furthermore, the variables used are most often restricted to the individual level (i.e. the micro-level), such as age, sex, and demographic and socioeconomic factors. Most likely, this has undercompensated insurers for high-risk individuals and therefore micro-level information alone does not predict healthcare utilization in a satisfactory manner.

The risk equalization models used in Belgium and the Netherlands include an environmental variable in addition to the standard demographic and prior utilization variables, i.e. degree of urbanization, while the Swiss model includes ‘region’ (Van der Veen 2007). However, no country uses neighborhood characteristics as risk adjusters. Van de Ven et al. (2013) conclude that prediction models of Switzerland, Belgium, the Netherlands, Israel and Germany need further improvement: “Despite the improvements in the risk equalization formulas there are still many (sub)groups of high-risk people who are undercompensated for the basic health insurance” ([52], p., 240).

Neighborhood characteristics may help to fill this gap and increase the quality of the prediction models. Visser et al. [53] show that, although ‘regional effects’ in the risk equalization model were small, some improvements could be made by introducing ‘air pollution’ into the Dutch equalization model. Visser et al. however fail to explain why particular environmental factors were included in this experiment and others were not. For instance, why was ‘number of rental houses’ included and what does this indicate? Without a theoretical framework that explains how and why certain characteristic aspects of healthcare utilization are included, compensation may be adversely affected.

With the introduction of the ‘Neighborhood and healthcare utilization model’ and the overview of neighborhood variables that are relevant to need and utilization which we have presented, we systematize the search for demand-related neighborhood variables to improve the equalization model. Schokkaert and Van de Voore [54] have demonstrated the importance of disentangling demand from supply factors – or legitimate and illegitimate risk-adjusters in risk-adjustment models. Our concept model introduces a whole pool of new demand-related predictors and delivers background understanding on the associations. We propose to add neighborhood characteristics to the already existing rich set of data. Even though the importance of prediction of the neighborhood variables might be small (which has to be corroborated by future research) the neighborhood might be relevant because of interaction effects between neighborhood characteristics and individual characteristics.

We would like to use the variable ‘number of rental houses’ as an example to show the applicability of our model. The number of rental houses has improved the prediction of healthcare utilization in the Netherlands (Visser et al., 2016). Based on our model, we argue that the number of rental houses may be a proxy for household stability, the condition of the built environment, green space, socioeconomic status of the neighborhood, or resources and facilities. A neighborhood with a high proportion of rental houses may be home to more people who are likely to move house (because they are less bound to one particular house) and thus with higher residential mobility and instability. Furthermore, tenants may take less good care of buildings and the outside spaces (where present) than homeowners. They may have a lower average income. It is also likely that a high proportion of rental houses in a neighborhood is a proxy for particular resources that best suit the needs of the local population, such as particular healthcare amenities. In this case, the number of rental houses may relate to supply-side factors. The distinction between neighborhood characteristics and supply-side factors is critical. As mentioned previously, the inclusion of supply-side factors does not enhance a prediction model on ‘need’ and utilization.

We suggest that, instead of using the black box variable ‘number of rental houses’, variables relating to stability, upkeep of the built environment, green space and the socioeconomic status of the neighborhood may improve the prediction of healthcare utilization. Moreover, based on Additional files 1 and 2, we would like to suggest more neighborhood characteristics as determinants for predicting utilization, and most probably also predicting wider healthcare expenditure.

## Conclusions

This paper shows that it is theoretically reasonable to use physical and social neighborhood characteristics to predict healthcare utilization and costs, because empirical evidence reveals that physical and social neighborhood characteristics are associated with health outcomes and mortality, either directly (via physiological pathways) or via health mediators (i.e., via psychological pathways and health behavioral pathways). Furthermore, physical and social neighborhood characteristics have been associated with healthcare utilization, although less comprehensive evidence is available for these associations. Based on our model and the supporting international literature, and despite the current lack of proof of the causal link between neighborhood characteristics and healthcare utilization, we would suggest testing the importance of neighborhood characteristics for prediction models, because there is an urgent need to further improve the prediction of healthcare utilization and costs. Experiments with neighborhood characteristics in the risk equalization model of the Dutch healthcare system have produced promising results. We are convinced that our concept model is applicable in other countries as well and will contribute to more demand-related neighborhood characteristics in prediction models.

## Additional files

Additional file 1: Overview of literature on physical neighborhood environmental characteristics in relation to mediators, health outcomes and healthcare utilization. (DOCX 31 kb)

Additional file 2: Overview of literature of social neighborhood environmental characteristics in relation to mediators, health outcomes and healthcare utilization. (DOCX 27 kb)

## Abbreviation

PA: Physical activity

## Footnotes

1 Although food is produced mostly outside the neighborhood, the food supply is close by and neighborhoods vary in access to healthy food; some neighborhoods are even called ‘food-desserts’ (Beaulac et al. 2009).

2 “The theory of the diffusion of innovations suggests that innovative behaviors (e.g., use of preventive services) diffuse much more rapidly in communities that are cohesive and in which members know and trust one another.” Kawachi, 1999, page 1190.
