# Supplementary material for: Neighborhood characteristics as determinants of healthcare utilization – a theoretical model
Source: Health Econ Rev. 2019 Mar 6;9:7. doi: 10.1186/s13561-019-0226-x (PMC6734422; doi:10.1186/s13561-019-0226-x)
Supplement: Supplementary file 1 — Overview of literature on physical neighborhood environmental characteristics in relation to mediators, health outcomes and healthcare utilization. (DOCX 31 kb) [file 13561_2019_226_MOESM1_ESM.docx]

**Additional file 1**

**Online Appendix 1**

Overview of literature on physical neighborhood environmental characteristics in relation to mediators, health outcomes and healthcare utilization.

| **Physical neighborhood characteristics** | **Study outcomes** | | | |
| --- | --- | --- | --- | --- |
|  | **Mediators**^[[1]](#footnote-1)^ | **Self-perceived health / mental health**^[[2]](#footnote-2)^**/ well-being** | **Diseases & mortality** | **Healthcare utilization** |
| **Degree of urbanization**  Density, land use mix, sprawl, Urbanity | Land use mix and high density seems to encourage transit use for work and shopping trips (1). A review showed that in North America (but not in other continents) land use mix and sprawl is associated with weight status (2). Systematic review and meta-analysis on elderly showed evidence for an association of density/urbanity and total walking for transport (3). Residents of sprawling counties were likely to walk less during leisure time, weight more, and have greater prevalence of hypertension than residents of compact counties (4). | A review among elderly concluded that higher density of older adults is associated with better mental health for elderly (5). | The degree of urbanization is positively associated with premature mortality (< 65) (6).  Urbanization associated with homicide for both Black and White males in the US (7). | Some studies point towards less healthcare being used in rural areas (8). Other studies find that more healthcare is being used in rural areas (9) and other studies find no difference between rural and urban neighborhoods in healthcare utilization, like for example a study on dental healthcare utilization (10). |
| **Public and open space;**  **(the maintenance of the) built environment.**  Walkability & bikeability, pedestrian and cycling infrastructure^[[3]](#footnote-3)^, public transportation, street/neighborhood design, other features of urban design, street connectivity, public buildings, areas  characterized by derelict  buildings and abundant  graffiti, open public  spaces and few buffers  between public and  private spaces, traffic safety, littering, vandalism, maintenance, cleanliness, condition of private and public buildings and streets. | A review concluded that walkable neighborhoods are associated with increased PA, lower overweight, and less alcohol abuse (11). A review on the built environment and PA stated that all studies show expected direction of association or null (12). A review about elderly concluded that PA resources are associated with PA and change in PA and more accessible neighborhood design supported greater levels of walking of elderly (5). A meta-analysis on elderly showed an association with littering, vandalism, decay and walkability for transportation (3). Review on adolescents: only one study showed an association between walkability and accelerometer-based PA (13).  Urban design can facilitate PA (14, 15). A South African study on adults showed that a pleasant scenery^[[4]](#footnote-4)^ were associated with walking for leisure (16). (Increased) Traffic safety in neighborhoods associated with being active (17). Neighborhood disadvantage (e.g. abandoned buildings, graffiti, crime) was associated with drug use (18). | One article of the Mair et al. review (19) showed an association between neighborhood walkability and depressive symptoms (20). Two other articles on public space showed also associations with depression (21, 22).  Living in a place where the sidewalks are in a good condition was associated with better subjective social life in Uruguay (23). | Conclusions of a systematic review: High walkable environment, fast food restaurants, supermarket/grocery stores were associated with blood pressure, body mass index, diabetes mellitus and metabolic syndrome. High density traffic, road proximity and fast food restaurants were associated with cardiovascular disease (24).  Canadian elderly living in a less than ideally maintained physical environment (deficits in cleanliness, neatness and repairs needed inside and outside house) demonstrated a higher risk of death five years later (25). | A Study from South Korea showed that the utilization of the senior centers (offering preventive medical consultations and health promotion programs) was affected by convenience of transportation and total travel time to the facilities (26).  An Australian study in 88 neighborhoods showed: higher walkability associated with less hospital admissions and lower hospital costs (27).  A Canadian study showed, elderly living in a less than ideally maintained physical environment demonstrated higher risk for an institutionalization (e.g. nursing home) (25). |
| **Resources and facilities:** public services, healthcare, healthy food, schools, playgrounds, commercial functions, and recreational opportunities | A review concluded that the availability of PA equipment is associated with vigorous PA and sports, while the connectivity of trails is associated with active commuting (28). A systematic review and meta-analysis on elderly’s total walking for transportation showed evidence for a positive association with access to services and destinations (3). | One British study showed that perceived good quality of facilities in the area (social/leisure, facilities for people aged 65+, rubbish collection, health services, transport, closeness to shops, somewhere nice to walk) is associated with good health and functioning (29).  In Uruguay access to electricity was positively associated with subjective health overall happiness indicators (23) | An US study on adults showed an association between neighborhood resources and facilities (including food stores and physical activity resources) with cardiovascular health (30). | Older U.S. adults living in a county with a higher dentist-to-population ratio were more likely to use dental services (31). |
| **Green space and blue space.** (other terms used: Greenness, green environment, physical green, urban green space, urban design, physical change to the green environment, residential greenness, access to nature and green space, urban forests) | Reviews concluded that the evidence about green space facilitating PA remains weak and effect sizes small (15, 32).  A review reported mixed evidence of association between greenspace and BMI (32). Another review concluded that physical change to the green environment combined with PA programs increases PA (Hunter et al., 2015). | A review concluded that there is limited evidence for the relationship surrounding greenness and mental health in adults (33). Another review concluded that there is weak evidence for urban links between green space and physical and mental health and well-being (15).  Blue space is related to self-perceived health (34) and mental health (35). | A review concluded that in areas with higher residential greenness the risk of cardiovascular disease mortality was lower (36). A review on green space and obesity reports found some associations between green space an cardiovascular disease and diabetes (32).  Green environments were not associated with reduced mortality in a US study (37) or less cardiovascular disease in Australia (38). | In Catalonia (Spain), green but not blue space showed to be associated with visits to mental health specialists and intake of mental health medication (39). Korea: urban areas with larger forests had lower medical care use (40). |
| **Environmental noise;** Main burden of environmental noise is related to traffic noise. Noise from road, rail, air traffic, industrial, wind turbines  environmental noise, transportation noise, decibel, L_DEN_, L_night_. Equivalent noise levels | Traffic noise is associated with sleep disturbance (41, 42) and annoyance (43). Traffic and aircraft noise increase risk of hypertension (44-47), cognitive responses such as task interference (43), and effects on children’s learning (48). Aircraft noise seems to be worse for cognition of children than traffic noise (45). Environmental noise is associated with immune system deficiencies (42) and stress hormone changes (49, 50). | A review (51) showed: environmental noise is associated with mental health symptoms such as depression and anxiety but not with impaired psychological functioning. Chronic aircraft noise exposure in children impairs quality of life but does not lead to depression or anxiety. A more recent review on children showed again, no serious mental health problems due to noise, but there is growing evidence for an association with increased hyperactivity symptoms (42) | Traffic noise increases the risk of ischemic heart disease, including myocardial infarction and stroke (44, 46, 52), type 2 diabetes (53, 54), hearing loss (42) , and mortality (55). | Aircraft noise is associated with and increased use of anxiolytics medication; in some countries also the use of antihypertensive medication (56). |
| **Air pollution**  Air quality, particulate air pollution, particulate matter (PM), nitrogen dioxide ozone, road traffic, local emissions, emissions from woodstove into the ambient air, smog (= short-term exposure) living in polluted areas (= long-term exposure). | Elderly (vulnerable) young children (developing airways) are at highest risk for respiratory and cardiovascular effects (57).  Air pollution is associated with school absentism (57, 58) and cognitive effects (57). | The WHO report 2013 refers to an effect of air pollution on health effects in general (WHO, 2013).  Limited evidence has been published suggesting that the nitrogen dioxide can enhance allergic  responses (57). | Traveling in traffic and living in busy streets has been associated with increased risks of morbidity and mortality(57).  Air pollution is associated with numerous health outcomes: birth outcomes (59), respiratory and cardiovascular diseases, lung cancer and mortality (57, 58). PM2.5 and ozone at concentrations below current US standards showed higher risks of mortality for racial minorities and people with low income (60). | Premature birth (57) and higher hospital admission for respiratory and cardiovascular symptoms in areas with higher level of nitrogen dioxide (61). |

## References Appendix 1

1. Frank LD, Pivo G. Impacts of Mixed Use and Density on Utilization of Three Modes of Travel: Single-occupant Vehicle, Transit, and Walking. Transportation Research Record. 1995;1466:44-52.

2. Mackenbach JD, Rutter H, Compernolle S, Glonti K, Oppert JM, Charreire H, et al. Obesogenic environments: a systematic review of the association between the physical environment and adult weight status, the SPOTLIGHT project. BMC Public Health. 2014;14:233.

3. Cerin E, Nathan A, van Cauwenberg J, Barnett DW, Barnett A, Older Adults working group CEPA. The neighbourhood physical environment and active travel in older adults: a systematic review and meta-analysis. Int J Behav Nutr Phys Act. 2017;14(1):15.

4. Ewing R, Schmid T, Killingsworth R, Zlot A, Raudenbush S. Relationship between urban sprawl and physical activity, obesity, and morbidity. Am J Health Promot. 2003;18(1):47-57.

5. Yen IH, Michael YL, Perdue L. Neighborhood environment in studies of health of older adults: a systematic review. Am J Prev Med. 2009;37(5):455-63.

6. Reijneveld SA, Verheij RA, de Bakker DH. Relative importance of urbanicity, ethnicity and socioeconomic factors regarding area mortality differences. J Epidemiol Community Health. 1999;53(7):444.

7. Cubbin C, Pickle LW, Fingerhut L. Social context and geographic patterns of homicide among US black and white males. Am J Public Health. 2000;90(4):579-87.

8. Coughlin SS, Thompson TD. Colorectal cancer screening practices among men and women in rural and nonrural areas of the United States, 1999. The Journal of Rural Health. 2004;20(2):118-24.

9. Chan L, Wang H, Terdiman J, Hoffman J, Ciol MA, Lattimore BF, et al. Disparities in outpatient and home health service utilization following stroke: results of a 9-year cohort study in Northern California. PM&R. 2009;1(11):997-1003.

10. Lang I, Gibbs S, Steel N, Melzer D. Neighbourhood deprivation and dental service use: a cross-sectional analysis of older people in England. Journal of public health. 2008;30(4):472-8.

11. Renalds A, Smith TH, Hale PJ. A systematic review of built environment and health. Fam Community Health. 2010;33(1):68-78.

12. McCormack G, Shiell A. In search of causality: a systematic review of the relationship between the built environment and physical activity among adults. Int J Behav Nutr Phys Act. 2011;8(1):125.

13. De Meester F, Van Dyck D, De Bourdeaudhuij I, Deforche B, Sallis JF, Cardon G. Active living neighborhoods: is neighborhood walkability a key element for Belgian adolescents? BMC Public Health. 2012;12:7.

14. Hunter RF, Christian H, Veitch J, Astell-Burt T, Hipp JA, Schipperijn J. The impact of interventions to promote physical activity in urban green space: a systematic review and recommendations for future research. Soc Sci Med. 2015;124:246-56.

15. Lee AC, Maheswaran R. The health benefits of urban green spaces: a review of the evidence. Journal of Public Health. 2011;33(2):212-22.

16. Malambo P, Kengne AP, Lambert EV, De Villers A, Puoane T. Association between perceived built environmental attributes and physical activity among adults in South Africa. BMC Public Health. 2017;17(1):213.

17. Jongeneel-Grimen B, Busschers W, Droomers M, van Oers HA, Stronks K, Kunst AE. Change in neighborhood traffic safety: does it matter in terms of physical activity? PLoS One. 2013;8(5):e62525.

18. Storr CL, Chen CY, Anthony JC. "Unequal opportunity": neighbourhood disadvantage and the chance to buy illegal drugs. J Epidemiol Community Health. 2004;58(3):231-7.

19. Mair C, Diez Roux AV, Galea S. Are neighbourhood characteristics associated with depressive symptoms? A review of evidence. J Epidemiol Community Health. 2008;62(11):940-6, 8 p following 6.

20. Berke EM, Gottlieb LM, Moudon AV, Larson EB. Protective association between neighborhood walkability and depression in older men. J Am Geriatr Soc. 2007;55(4):526-33.

21. Hadley-Ives E, Stiffman AR, Elze D, Johnson SD, Dore P. Measuring neighborhood and school environments perceptual and aggregate approaches. J Hum Behav Soc Environ. 2000;3(1):1-28.

22. Weich S, Blanchard M, Prince M, Burton E, Erens B, Sproston K. Mental health and the built environment: cross-sectional survey of individual and contextual risk factors for depression. The British Journal of Psychiatry. 2002;180(5):428-33.

23. Gandelman N, Piani G, Ferre Z. Neighborhood determinants of quality of life. Journal of Happiness Studies. 2012;13(3):547-63.

24. Malambo P, Kengne AP, De Villiers A, Lambert EV, Puoane T. Built Environment, Selected Risk Factors and Major Cardiovascular Disease Outcomes: A Systematic Review. PLoS One. 2016;11(11):e0166846.

25. Wentzel C, Rose H, Rockwood K. Measurement of the influence of the physical environment on adverse health outcomes: technical report from the Canadian Study of Health and Aging. Int Psychogeriatr. 2001;13 Supp 1:215-21.

26. Kim HS, Miyashita M, Harada K, Park JH, So JM, Nakamura Y. Psychological, social, and environmental factors associated with utilization of senior centers among older adults in Korea. J Prev Med Public Health. 2012;45(4):244-50.

27. Yu Y, Davey R, Cochrane T, Learnihan V, Hanigan IC, Bagheri N. Neighborhood walkability and hospital treatment costs: A first assessment. Prev Med. 2017;99:134-9.

28. Wendel‐Vos W, Droomers M, Kremers S, Brug J, Van Lenthe FJ. Potential environmental determinants of physical activity in adults: a systematic review. Obes Rev. 2007;8(5):425-40.

29. Bowling A, Barber J, Morris R, Ebrahim S. Do perceptions of neighbourhood environment influence health? Baseline findings from a British survey of aging. J Epidemiol Community Health. 2006;60(6):476-83.

30. Unger E, Diez-Roux AV, Lloyd-Jones DM, Mujahid MS, Nettleton JA, Bertoni A, et al. Association of neighborhood characteristics with cardiovascular health in the multi-ethnic study of atherosclerosis. Circ Cardiovasc Qual Outcomes. 2014;7(4):524-31.

31. Lee W, Kim SJ, Albert JM, Nelson S. Community factors predicting dental care utilization among older adults. J Am Dent Assoc. 2014;145(2):150-8.

32. Lachowycz K, Jones AP. Greenspace and obesity: a systematic review of the evidence. Obes Rev. 2011;12(5):e183-e89.

33. Gascon M, Triguero-Mas M, Martinez D, Dadvand P, Forns J, Plasencia A, et al. Mental health benefits of long-term exposure to residential green and blue spaces: a systematic review. Int J Environ Res Public Health. 2015;12(4):4354-79.

34. Wheeler BW, White M, Stahl-Timmins W, Depledge MH. Does living by the coast improve health and wellbeing? Health Place. 2012;18(5):1198-201.

35. de Vries S, Ten Have M, van Dorsselaer S, van Wezep M, Hermans T, de Graaf R. Local availability of green and blue space and prevalence of common mental disorders in the Netherlands. BJPsych Open. 2016;2(6):366-72.

36. Gascon M, Triguero-Mas M, Martinez D, Dadvand P, Rojas-Rueda D, Plasencia A, et al. Residential green spaces and mortality: A systematic review. Environ Int. 2016;86:60-7.

37. Richardson EA, Mitchell R, Hartig T, de Vries S, Astell-Burt T, Frumkin H. Green cities and health: a question of scale? J Epidemiol Community Health. 2012;66(2):160-5.

38. Pereira G, Foster S, Martin K, Christian H, Boruff BJ, Knuiman M, et al. The association between neighborhood greenness and cardiovascular disease: an observational study. BMC Public Health. 2012;12:466.

39. Triguero-Mas M, Dadvand P, Cirach M, Martinez D, Medina A, Mompart A, et al. Natural outdoor environments and mental and physical health: relationships and mechanisms. Environ Int. 2015;77:35-41.

40. Lee K-S, Lee J-S, Kwon J-H. The effects of urban forests on the medical care use for respiratory disease in Korea: a structural equation model approach. International Journal of Public Policy. 2014;10(4-5):195-208.

41. Muzet A. Environmental noise, sleep and health. Sleep Med Rev. 2007;11(2):135-42.

42. Basner M, Babisch W, Davis A, Brink M, Clark C, Janssen S, et al. Auditory and non-auditory effects of noise on health. The Lancet. 2014;383(9925):1325-32.

43. Miedema H, Oudshoorn C. Annoyance from transportation noise: relationships with exposure metrics DNL and DENL and their confidence intervals. Environ Health Perspect. 2001;109(4):409.

44. Babisch W. Transportation noise and cardiovascular risk: updated review and synthesis of epidemiological studies indicate that the evidence has increased. Noise and Health. 2006;8(30):1-29.

45. WHO. Burden of disease from environmental noise - Quantification of healthy life years lost in Europe. Bonn: WHO European Centre for Environment and Health; 2011.

46. Van Kempen EMM, Kruize H, Boshuizen HC, Ameling CB, Staatsen BAM, de Hollander AEM. The association between noise exposure and blood pressure and ischemic heart disease: a meta-analysis. Environ Health Perspect. 2002;110(3):307.

47. Van Kempen EMM, Babisch W. The quantitative relationship between road traffic noise and hypertension: a meta-analysis. J Hypertens. 2012;30(6):1075-86.

48. Hygge S, Evans GW, Bullinger M. A prospective study of some effects of aircraft noise on cognitive performance in schoolchildren. Psychol Sci. 2002;13(5):469-74.

49. Babisch W. Stress hormones in the research on cardiovascular effects of noise. Noise and health. 2003;5(18):1-11.

50. Maschke C, Harder J, Ising H, Hecht K, Thierfelder W. Stress hormone changes in persons exposed to simulated night noise. Noise and Health. 2002;5(17):35.

51. Stansfeld S, Haines M, Burr M, Berry B, Lercher P. A review of environmental noise and mental health. Noise and Health. 2000;2(8):1.

52. Dzhambov AM, Dimitrova DD. Exposure-response relationship between traffic noise and the risk of stroke: a systematic review with meta-analysis. Archives of industrial hygiene and toxicology. 2016;67(2):136-51.

53. Dzhambov AM. Long-term noise exposure and the risk for type 2 diabetes: A meta-analysis: Erratum. Noise & health. 2015;17(75):123.

54. Sørensen M, Andersen ZJ, Nordsborg RB, Becker T, Tjønneland A, Overvad K, et al. Long-term exposure to road traffic noise and incident diabetes: a cohort study. Environ Health Perspect. 2013;121(2):217.

55. Halonen JI, Blangiardo M, Toledano MB, Fecht D, Gulliver J, Ghosh R, et al. Is long-term exposure to traffic pollution associated with mortality? A small-area study in London. Environmental Pollution. 2016;208:25-32.

56. Floud S, Vigna-Taglianti F, Hansell A, Blangiardo M, Houthuijs D, Breugelmans O, et al. Medication use in relation to noise from aircraft and road traffic in six European countries: results of the HYENA study. Occup Environ Med. 2011;68(7):518-24.

57. WHO. Review of evidence on health aspects of air pollution – REVIHAAP Project Technical Report. Bonn: The WHO European Centre for Environment and Health; 2013.

58. Héroux ME, Anderson HR, Atkinson R, Brunekreef B, Cohen A, Forastiere F, et al. Quantifying the health impacts of ambient air pollutants: recommendations of a WHO/Europe project. Int J Public Health. 2015;60(5):619-27.

59. Sram RJ, Binkova B, Dejmek J, Bobak M. Ambient air pollution and pregnancy outcomes: a review of the literature. Environ Health Perspect. 2005;113(4):375-82.

60. Di Q, Wang Y, Zanobetti A, Wang Y, Koutrakis P, Choirat C, et al. Air Pollution and Mortality in the Medicare Population. N Engl J Med. 2017;376(26):2513-22.

61. Dijkema MBA, van Strien RT, van der Zee SC, Mallant SF, Fischer P, Hoek G, et al. Spatial variation in nitrogen dioxide concentrations and cardiopulmonary hospital admissions. Environ Res. 2016;151:721-7.

1. Mediators were health-related behavior, stress-level, eating habits, participation, and willingness to use healthcare. [↑](#footnote-ref-1)
2. Mental health = Self-perceived and objective diagnoses of mental health problems. [↑](#footnote-ref-2)
3. Pedestrian and cycling infrastructure includes presence, condition, and maintenance of sidewalks, bike lanes, cross walks, street lights, traffic lights. [↑](#footnote-ref-3)
4. E.g., sidewalks shade from trees, traffic, and well-lit streets. [↑](#footnote-ref-4)
