# Supplementary material for: Neighborhood characteristics as determinants of healthcare utilization – a theoretical model
Source: Health Econ Rev. 2019 Mar 6;9:7. doi: 10.1186/s13561-019-0226-x (PMC6734422; doi:10.1186/s13561-019-0226-x)
Supplement: Supplementary file 2 — Overview of literature of social neighborhood environmental characteristics in relation to mediators, health outcomes and healthcare utilization. (DOCX 27 kb) [file 13561_2019_226_MOESM2_ESM.docx]

## Additional file 2

## Online Appendix 2: Overview of literature of social neighborhood environmental characteristics in relation to mediators, health outcomes and healthcare utilization.

| **Social neighborhood characteristics** | **Study outcome** | | | |
| --- | --- | --- | --- | --- |
|  | **Mediators**^[[1]](#footnote-1)^ | **Self-perceived health / mental health**^[[2]](#footnote-2)^**/ well-being** | **Diseases & mortality** | **Healthcare utilization** |
| **Social capital**  Articles about social capital encompass measures of social capital, social participation, social cohesion, (dis)organization of communities, informal social control, , attachment to the neighborhood, degree and nature of social connections between neighbors, social organization, and clubs; social norms, culture  church, ethnical background, reputation, neighborliness, collective efficacy. | A review concluded that there is limited evidence that greater social capital and collective efficacy are associated with healthier weight status [1]. Another review’s findings about elderly on  neighborhood social environment and PA were inconsistent [2]. In the Netherlands: neighborhood social capital is associated with more PA and less smoking. Not associated with nutrition and sleep habits or moderate alcohol intake [3]. Improvements in neighborhood social cohesion were positively associated with PA [4]. | A review concluded that greater social capital is protective against depression [5,6]. A review about elderly concluded that findings on neighborhood social environment and self-reported health and mental health are inconsistent [2]. Lower social capital is associated with worse self-perceived health [6]. | Neighborhood cohesion is associated with lower cardiovascular disease [6].  A review about elderly concluded that neighborhood social environment was associated with heart disease and mortality [2]. | More perceived social capital is associated with more preventive dental visits in the US [7]. More social cohesion is related to the use of more health services [8] and more cancer screening [9]. Others found no association between the social environment and healthcare use, like accident and emergency department attendance rates of children [10]. |
| **Crime and violence**  levels of safety and violence, neighborhood problems: drug use, drug dealing, shootings, murder, abandoned buildings, neighbors on welfare, homeless people and prostitution on the street; perception of violence, perceptions of neighborhood environment. | A review concluded that there is no evidence of an association between neighborhood problems and PA among elderly [2]. The perception of unsafe neighborhoods was associated with lower PA, especially for women, living in urban low-income housing [11]. Police-recorded crime on the neighborhood level was associated with stress for African-American and Latina women in a US diabetes population [12].  In the Netherlands improvements between 2006 and 2009 with respect to social and physical disorder were associated with increasing PA [4]. | A review concluded that disorder seems to affect depression via neighborhood perception [5]. Violence and hazardous conditions could be associated with increased depressive symptoms [6].  Neighborhood problems were associated with self-rated health of elderly in Britain [13]. | The poorest Canadian neighborhood with high crime rates showed higher risk for HIV infections among a cohort of drug users [14].  Public injecting might be associated with higher risk of HIV [15]. | UK: No significant association between wards with higher violent crime rates and children’s admission to the accident and emergency hospital department, [10]. Canada: Neighborhood problems were not associated with utilization for child injuries [16] but higher perceived neighborhood disorder was associated with higher rates of total health services usage (specialist / emergency room visits)  [17]. Paris: neighborhood safety was associated with lower overdue cervical cancer screening rates among women, but no association was found with neighborhood disorder [18].  USA: Elderly women living in areas with higher crime rates are less likely to use mammography [19]. Neighborhood problems are associated with higher community-based mental health service costs and increased hospital-based mental health service costs for adults with a chronic mental illness [20]. Neighborhood safety was not associated with the use of primary care use in NYC [21]. |
| **Socioeconomic status of the neighborhood**  Inequality, resources, wealth, deprivation, neighborhood disadvantage | A review concluded that neighborhoods with lower SES generally show more smoking, dietary fat consumption, alcohol consumption, and violence [22]. Neighborhood disadvantage is associated with drug use [23,24]. | A review reported that thirteen out of the twenty-five studies that examined the effect of neighborhood socioeconomic position on depressive symptoms supported the presence of an association after adjustment for  individual-level characteristics [5]. A review about elderly also found that low economic status of the neighborhood is associated with poor health [2]. | A review found that more deprived neighborhoods have an increased risk of mortality [22]. One study reports that deprived neighborhoods experience higher homicide rates [25]. Neighborhood deprivation independently influences injury risk of children [26]. Neighborhood deprivation might be associated with higher risk of HIV [15]. | In neighborhoods with lower socioeconomic status *more* [27,28,26] or *less* [29,30] healthcare is being used compared with areas with a higher socioeconomic status; and some studies report no association [17,31,32]. |
| **Stability of the neighborhood**  residential (in-)stability, mobility | In Philadelphia a stable neighborhood could weaken the unfavorable association between stress and health  [33]. | Four of the eight studies that examined the association between residential mobility and depression found evidence of an association [5]. A French study showed an association between residential stability and self-reported health [34]. | Neighborhood residential instability was weakly associated with ischemic heart disease as well as shorter survival time after myocardial infarction [35]. | Residential stability in Canadian neighborhoods increased health service use of patients with mental health disorders [29]. |
| **Ethnic composition**  Racial composition, racial heterogeneity, Residential racial segregation, ethnic enclaves, | A study on older Chinese and Hispanic immigrants to the U.S. showed an association between increase in BMI (during 9 years) and a decrease in  neighborhood co-ethnic concentration, mainly for the Chinese population [36]. | A review summarized that only four of the ten studies that examined racial/ethnic composition of neighborhoods found an association with depression [5]. The relationship between residential segregation and subjective rated health of Latinos living in Washington State depends on the level and change of segregation [37]. A review among elderly concluded that while ethnic enclaves for Latinos seems to be beneficial for health (depression and self-related health), this does not work for African American elderly [2]. | A review concluded that elderly Latino’s experience an ethnic enclave advantage regarding morbidity levels [2]. | Gaskin et al. [38] found that disparities in healthcare utilization are related to both individuals’ racial and ethnic identity and the racial and ethnic composition of their communities. |

## References Appendix 2

1. Glonti, K., Mackenbach, J.D., Ng, J., Lakerveld, J., Oppert, J.M., Bardos, H., McKee, M., Rutter, H.: Psychosocial environment: definitions, measures and associations with weight status – a systematic review. Obes. Rev. **17**(1), 81-95 (2016).

2. Yen, I.H., Michael, Y.L., Perdue, L.: Neighborhood environment in studies of health of older adults: a systematic review. Am. J. Prev. Med. **37**(5), 455-463 (2009). doi:10.1016/j.amepre.2009.06.022

3. Mohnen, S.M., Volker, B., Flap, H., Groenewegen, P.P.: Health-related behavior as a mechanism behind the relationship between neighborhood social capital and individual health--a multilevel analysis. BMC Public Health **12**, 116 (2012). doi:10.1186/1471-2458-12-116

4. Jongeneel-Grimen, B., Droomers, M., van Oers, H.A., Stronks, K., Kunst, A.E.: The relationship between physical activity and the living environment: a multi-level analyses focusing on changes over time in environmental factors. Health Place **26**, 149-160 (2014). doi:10.1016/j.healthplace.2013.12.003

5. Mair, C., Diez Roux, A.V., Galea, S.: Are neighbourhood characteristics associated with depressive symptoms? A review of evidence. J. Epidemiol. Community Health **62**(11), 940-946, 948 p following 946 (2008). doi:10.1136/jech.2007.066605

6. Diez Roux, A.V., Mair, C.: Neighborhoods and health. Ann. N. Y. Acad. Sci. **1186**, 125-145 (2010). doi:10.1111/j.1749-6632.2009.05333.x

7. Iida, H., Rozier, R.G.: Mother-perceived social capital and children's oral health and use of dental care in the United States. Am. J. Public Health **103**(3), 480-487 (2013). doi:10.2105/AJPH.2012.300845

8. Nguyen, D.D., Ho, K.H., Williams, J.H.: Social determinants and health service use among racial and ethnic minorities: Findings from a community sample. Soc. Work Health Care **50**(5), 390-405 (2011).

9. Leader, A.E., Michael, Y.L.: The association between neighborhood social capital and cancer screening. Am. J. Health Behav. **37**(5), 683-692 (2013). doi:10.5993/AJHB.37.5.12

10. Kendrick, D., Mulvaney, C., Burton, P., Watson, M.: Relationships between child, family and neighbourhood characteristics and childhood injury: a cohort study. Soc. Sci. Med. **61**(9), 1905-1915 (2005). doi:10.1016/j.socscimed.2005.04.003

11. Bennett, G.G., McNeill, L.H., Wolin, K.Y., Duncan, D.T., Puleo, E., Emmons, K.M.: Safe to walk? Neighborhood safety and physical activity among public housing residents. PLoS Med. **4**(10), 1599-1606 (2007). doi:10.1371/journal.pmed.0040306

12. Tamayo, A., Mujahid, M.S., Laraia, B., Warton, E.M., Blanchard, S.D., Kelly, M., Moffet, H.H., Adler, N., Schillinger, D., Karter, A.J.: Police-Recorded Crime and Perceived Stress among Patients with Type 2 Diabetes: the Diabetes Study of Northern California (DISTANCE). J. Urban Health **93**(5), 745-757 (2016). doi:10.1007/s11524-016-0069-2

13. Bowling, A., Barber, J., Morris, R., Ebrahim, S.: Do perceptions of neighbourhood environment influence health? Baseline findings from a British survey of aging. J. Epidemiol. Community Health **60**(6), 476-483 (2006). doi:10.1136/jech.2005.039032

14. Maas, B., Fairbairn, N., Kerr, T., Li, K., Montaner, J.S., Wood, E.: Neighborhood and HIV infection among IDU: place of residence independently predicts HIV infection among a cohort of injection drug users. Health Place **13**(2), 432-439 (2007). doi:10.1016/j.healthplace.2006.05.005

15. Tempalski, B., McQuie, H.: Drugscapes and the role of place and space in injection drug use-related HIV risk environments. Int. J. Drug Policy **20**(1), 4-13 (2009). doi:10.1016/j.drugpo.2008.02.002

16. Soubhi, H., Raina, P., Kohen, D.: Neighborhood, family, and child predictors of childhood injury in Canada. Am. J. Health Behav. **28**(5), 397-409 (2004).

17. Martin-Storey, A., Temcheff, C.E., Ruttle, P.L., Serbin, L.A., Stack, D.M., Schwartzman, A.E., Ledingham, J.E.: Perception of neighborhood disorder and health service usage in a Canadian sample. Ann. Behav. Med. **43**(2), 162-172 (2012). doi:10.1007/s12160-011-9310-0

18. Rigal, L., Saurel-Cubizolles, M.-J., Falcoff, H., Bouyer, J., Ringa, V.: Do social inequalities in cervical cancer screening persist among patients who use primary care? The Paris Prevention in General Practice survey. Prev. Med. **53**(3), 199-202 (2011).

19. Mobley, L.R., Kuo, T.-M., Andrews, L.: How Sensitive Are Multilevel Regression Findings to Defined Area of Context? A Case Study of Mammography Use in California. Med. Care Res. Rev. **65**(3), 315–337 (2008).

20. Harkness, J., Newman, S.J., Salkever, D.: The Cost‐Effectiveness of Independent Housing for the Chronically Mentally Ill: Do Housing and Neighborhood Features Matter? Health Serv. Res. **39**(5), 1341-1360 (2004).

21. Ryvicker, M., Gallo, W.T., Fahs, M.C.: Environmental factors associated with primary care access among urban older adults. Soc. Sci. Med. **75**(5), 914-921 (2012).

22. Pickett, K.E., Pearl, M.: Multilevel analyses of neighbourhood socioeconomic context and health outcomes: a critical review. Journal of Epidemiology and Community Health **55**(2), 111-122 (2001). doi:10.1136/jech.55.2.111

23. Boardman, J.D., Finch, B.K., Ellison, C.G., Williams, D.R., Jackson, J.S.: Neighborhood disadvantage, stress, and drug use among adults. J. Health Soc. Behav. **42**(2), 151-165 (2001).

24. Nandi, A., Glass, T.A., Cole, S.R., Chu, H., Galea, S., Celentano, D.D., Kirk, G.D., Vlahov, D., Latimer, W.W., Mehta, S.H.: Neighborhood poverty and injection cessation in a sample of injection drug users. Am. J. Epidemiol. **171**(4), 391-398 (2010). doi:10.1093/aje/kwp416

25. Thompson, S.K., Gartner, R.: The spatial distribution and social context of homicide in Toronto’s neighborhoods. Journal of research in crime and delinquency **51**(1), 88-118 (2014).

26. Haynes, R., Reading, R., Gale, S.: Household and neighbourhood risks for injury to 5-14 year old children. Soc. Sci. Med. **57**(4), 625-636 (2003).

27. Durbin, A., Moineddin, R., Lin, E., Steele, L.S., Glazier, R.H.: Examining the relationship between neighbourhood deprivation and mental health service use of immigrants in Ontario, Canada: a cross-sectional study. BMJ Open **5**(3), e006690 (2015). doi:10.1136/bmjopen-2014-006690

28. Van der Linden, J., Drukker, M., Gunther, N., Feron, F., Van Os, J.: Children’s mental health service use, neighbourhood socioeconomic deprivation, and social capital. Soc. Psychiatry Psychiatr. Epidemiol. **38**(9), 507-514 (2003).

29. Ngamini Ngui, A., Perreault, M., Fleury, M.J., Caron, J.: A multi-level study of the determinants of mental health service utilization. Rev. Epidemiol. Sante Publique **60**(2), 85-93 (2012). doi:10.1016/j.respe.2011.09.007

30. Heaman, M.I., Green, C.G., Newburn-Cook, C.V., Elliott, L.J., Helewa, M.E.: Social inequalities in use of prenatal care in Manitoba. Journal of Obstetrics and Gynaecology Canada **29**(10), 806-816 (2007).

31. Ivert, A.-K., Levander, M.T., Merlo, J.: Adolescents' utilisation of psychiatric care, neighbourhoods and neighbourhood socioeconomic deprivation: a multilevel analysis. PLoS One **8**(11), e81127 (2013).

32. Drukker, M., Driessen, G., Krabbendam, L., Van Os, J.: The wider social environment and mental health service use. Acta Psychiatr. Scand. **110**(2), 119-129 (2004).

33. Matthews, S.A., Yang, T.C.: Exploring the role of the built and social neighborhood environment in moderating stress and health. Ann. Behav. Med. **39**(2), 170-183 (2010). doi:10.1007/s12160-010-9175-7

34. Debrand, T., Pierre, A., Allonier, C., Lucas-Gabrielli, V.: Critical urban areas, deprived areas and neighbourhood effects on health in France. Health Policy **105**(1), 92-101 (2012). doi:10.1016/j.healthpol.2012.01.001

35. Chaix, B., Rosvall, M., Merlo, J.: Neighborhood socioeconomic deprivation and residential instability: effects on incidence of ischemic heart disease and survival after myocardial infarction. Epidemiology **18**(1), 104-111 (2007). doi:10.1097/01.ede.0000249573.22856.9a

36. Le-Scherban, F., Albrecht, S.S., Osypuk, T.L., Sanchez, B.N., Diez Roux, A.V.: Neighborhood ethnic composition, spatial assimilation, and change in body mass index over time among Hispanic and Chinese immigrants: Multi-Ethnic Study of Atherosclerosis. Am. J. Public Health **104**(11), 2138-2146 (2014). doi:10.2105/AJPH.2014.302154

37. Plascak, J.J., Molina, Y., Wu-Georges, S., Idris, A., Thompson, B.: Latino residential segregation and self-rated health among Latinos: Washington State Behavioral Risk Factor Surveillance System, 2012-2014. Soc. Sci. Med. **159**, 38-47 (2016). doi:10.1016/j.socscimed.2016.04.035

38. Gaskin, D.J., Dinwiddie, G.Y., Chan, K.S., McCleary, R.: Residential segregation and disparities in health care services utilization. Med. Care Res. Rev. **69**(2), 158-175 (2012). doi:10.1177/1077558711420263

1. Mediators were health-related behavior, stress-level, eating habits, participation, and willingness to use healthcare. [↑](#footnote-ref-1)
2. Mental health = Self-perceived and objective diagnoses of mental health problems. [↑](#footnote-ref-2)
